# Simultaneous Determination of Coumarin and Its Derivatives in Tobacco Products by Liquid Chromatography-Tandem Mass Spectrometry

**DOI:** 10.3390/molecules21111511

**Published:** 2016-11-10

**Authors:** Zhiqin Ren, Bo Nie, Tong Liu, Fei Yuan, Feng Feng, Yuan Zhang, Weie Zhou, Xiuli Xu, Meiyi Yao, Feng Zhang

**Affiliations:** 1 Institute of Food Safety (Tobacco Safety and Control Technology Center), Chinese Academy of Inspection & Quarantine, Beijing 100176, China; zhiqin0513@126.com (Z.R.); liutongyes@163.com (T.L.); feyyuan@163.com (F.Y.); fengf_2005@163.com (F.F.); 13840149878@163.com (Y.Z.); zhouweietj@126.com (W.Z.); xuxiuli_78@163.com (X.X.); yaomeiyi124@126.com (M.Y.); 2 Key Laboratory of Chinese Internal Medicine of Ministry of Education and Beijing, Dongzhimen Hospital, Beijing University of Chinese Medicine, Beijing 100700, China; nieboww_1977@163.com

**Keywords:** high performance liquid chromatography-tandem mass spectrometry (HPLC-MS/MS), flavor additive, cigarette

## Abstract

In this paper an analytical method based on high performance liquid chromatography coupled to tandem mass spectrometry (HPLC-MS/MS) for the determination of coumarin and its derivatives in tobacco products was developed. The MS/MS fragmentation pathways of the eight coumarins were elucidated. The new analytical method was defined based on two main axes, an extraction procedure with acetonitrile and analyte detection performed by HPLC-MS/MS in electron impact mode. The excellent selectivity and sensitivity achieved in multiple reaction monitoring (MRM) mode allowed satisfactory confirmation and quantitation for the coumarin flavor additives. Under the optimized gradient elution conditions, it took only 4.5 min to separate all eight coumarins. Good linearity for all the analytes were confirmed by the correlation coefficient r^2^, ranging from 0.9987 to 0.9996. The limits of detection (LODs) and limits of quantitation (LOQs) of these compounds were in the range of 0.5–1.7 μg/kg and 1.7–5.2 μg/kg, respectively. The average recoveries at three spiked levels (LOQ, 1.5LOQ, 2LOQ) were all in the range of 69.6%–95.1% with RSDs (*n* = 6) lower than 5.3%. The method of HPLC-MS/MS developed in this study was initially applied to the research of coumarin flavor additives in tobacco products collected from the located market in Beijing from China and proved to be accurate, sensitive, convenient and practical.

## 1. Introduction

The WHO has estimated that tobacco use is currently responsibility for the death of about six million people across the world each year, with many of these deaths occurring prematurely [1]. In order to improve the physicochemical character and modify the basic taste of tobacco products, many kinds of flavor additives are used in the production process. These additives contribute distinctive and brand-specific sensory effects, so they are widely used in the tobacco industry [2,3]. In most countries it is now generally accepted that tobacco additives are associated with carcinogenic activity, mutagenicity, and hallucinogenic effects [4,5,6,7]. 

Coumarin, a phytochemical with a sweet herbaceous odor found in many plant species, has been used as a flavoring and fragrance enhancer [8]. It can greatly improve the attractiveness of food, cosmetic and tobacco products [9]. Coumarin has been shown to cause hepatoxicity in animals and in the USA its use as a food additive has been banned since 1956 [10]. The Framework Convention on Tobacco Control (FCTC) recommends that the coumarin should be restricted or banned [11,12,13]. Moreover, the German Tobacco Ordinance (Tabakverordnung) prohibits adding coumarin to tobacco since 1974 [14]. However, a variety of studies [15,16,17,18] have shown that 7-methylcoumarin, 7-methoxy-coumarin, 3,4-dihydrocoumarin, 7-ethoxy-4-methylcoumarin, pyranocoumarin, 7-diethylamino-coumarin and sincoumar (acenocoumarin) have been used as flavor additives to replace coumarin in certain foods and have been shown to have physiological toxicity. Animal experiments [19] have shown that these seven coumarin derivatives display moderate liver and kidney toxicity. Taking the side effects of coumarin derivatives into account, a rapid analytical method for the determination of coumarin derivatives in tobacco is necessary and critical.

So far, a variety of analytical methods based on GC [20,21], GC–MS [22], HPLC [23,24,25,26,27], and LC–MS [28,29] have been developed to analyze coumarins, but the low accuracy, high organic reagent consumption and long analysis times of these conventional methods are primary obstacles because of the complex matrix effects of coumarins. In recent years, tandem mass spectrometry (MS/MS) has been reported to provide a much higher degree of assurance than the single stage mass spectrometry technique when determining analytes in many complex matrix [30]. The application of multiple reactions monitoring (MRM) mode can provide a sensitive and selective gain because the fragmentation reaction implies two different characteristics of ion pairs for each target compound. To the best of our knowledge, there is no report of the use of HPLC-MS/MS for simultaneous determination of coumarin and its derivatives.

In this paper, we describe a new analytical method for simultaneous determination of coumarin and its derivatives in tobacco products by HPLC-MS/MS. Variables affecting the extraction and determination were optimized to achieve a better separation and recovery. The excellent selectivity and sensitivity achieved in MRM mode allowed satisfactory confirmation and quantitation for those target compounds. Additionally, the reliability and adaptability of the method were further verified by determination of linear range, recovery, and reproducibility with tobacco samples.

## 2. Results and Discussion

### 2.1. Optimization of Mass Spectrometry

In order to obtain the best mass spectra of the target analytes, the ESI–MS/MS data acquisition parameters was optimized. For this purpose, it is necessary to compromise between sensitivity and selectivity when choosing the appropriate MRM transitions.

**Figure 1:**
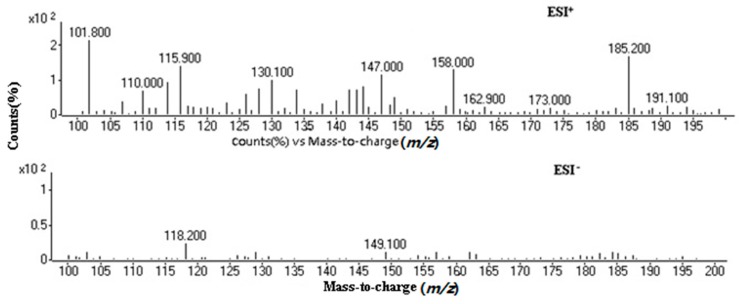
Precursor ions of coumarin in the ESI^+^/ESI^−^ mode.

Product spectra were acquired by collision-inducted dissociation (CID) with argon in product scan mode. The positive and negative modes were applied to select the most abundant *m*/*z* values for coumarin and its derivatives. The experimental results indicated that all target substances can give higher abundance precursor ions [M + H]^+^ in the ESI^+^ ion mode than in the ESI^−^ one under full scan (Figure 1). Therefore, the positive mode was selected for coumarin and its derivatives.

**Figure 2:**
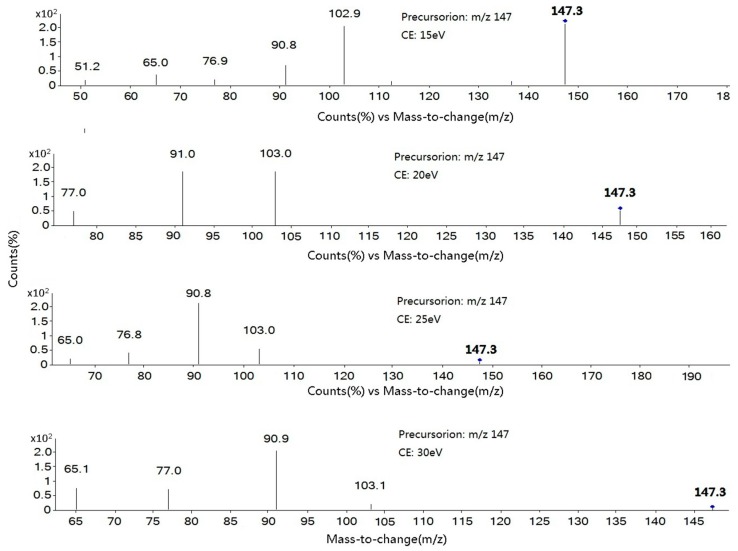
MS/MS spectra of coumarin using different collision energies (15, 20, 25 and 30 eV).

The collision energy (CE) is a critical parameter which affects sensitivity. Product ion scan mode data was acquired by collision-inducted dissociation (CID) for fragment ions. It is better to test three or four fragment ions for each analyte in samples like tobacco, and select two ions that are sensitive in the final MRM mode. Taking coumarin for example, the MS/MS spectra of the precursor ion (*m*/*z* 147.1) at 15, 20, 25 and 30 eV are given in Figure 2. The abundance of *m*/*z* 147 > *m*/*z* 91was increased from 50 cps to 250 cps in the range of 15 eV to 25 eV and was decreased from 250 cps to 200 cps in the range 25 eV to 30 eV. Therefore, 25 eV was chosen as the optimized CE value of *m*/*z* 147 > *m*/*z* 91 for coumarin. However, the precursor ion *m*/*z* 147 hardly yielded any *m*/*z* 103 product ion above 25 eV, so 15 eV was chosen as the optimized CE value of *m*/*z* 147 > *m*/*z* 103 for coumarin. According to the ion abundance of the optimized CE value, the *m*/*z* 91 ion with the largest abundance was chosen as the quantitative ion and ion *m*/*z* 103 as the qualitative one for coumarin. All the optimized MRM parameters, such as precursor ions and production ions are listed in Table 1.

**Figure 3:**
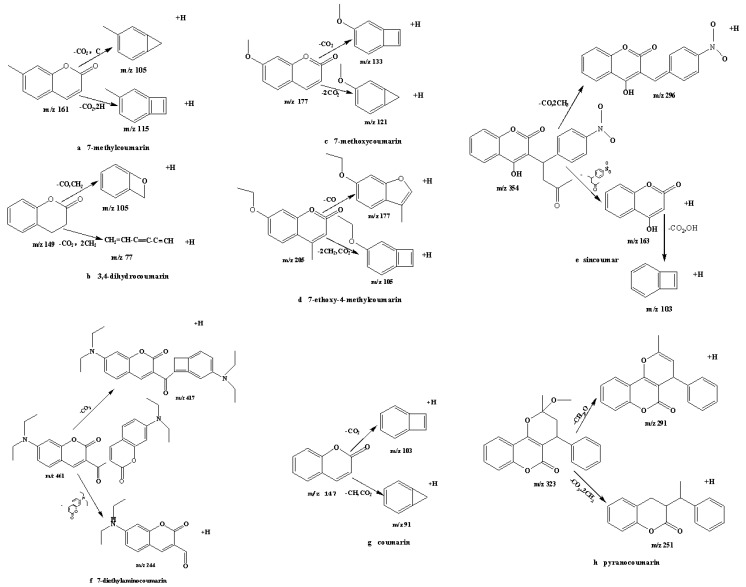
Fragmentation pathways of coumarin and its derivatives.

Attempts to deduce the identity of the ions have been made theoretically by sequential fragmentation. Coumarin can form a protonated molecular ion with *m*/*z* 147 [M + H]^+^ in the ESI^+^ ion mode. It formed *m*/*z* 103 fragment ions when it lost CO_2_ (*m*/*z* 44). Then it formed *m*/*z* 91 fragment ions when it lost both CO_2_ (*m*/*z* 44) and ·HC (*m*/*z* 13). Proposed fragmentation schemes are shown in Figure 3.

By analyzing the data in Table 1 and the structure of coumarin and its derivatives, the fragmentation mechanisms of the mass spectra of these compounds were studied. The similar fragments of 7-methylcoumarin (a), 7-methoxycoumarin (c), 7-diethylaminocoumarin (f) and coumarin (g) resulted from the consecutive loss of CO_2_ (*m*/*z* 44). For instance, the coumarin moiety (*m*/*z* 163) has a potential loss of·CO_2_ (*m*/*z* 44) to give a *m*/*z* 103 moiety. Similarly, 3,4-dihydrocoumarin (**b**), 7-ethoxy-4-methylcoumarin (**d**), and pyranocoumarin (**h**) readily lose CO_2_ (*m*/*z* 44) and C_2_H_4_ (*m*/*z* 28). In short, all the mechanisms above show the common fragmentation behavior of the coumarin and its derivatives.

**Figure 4:**
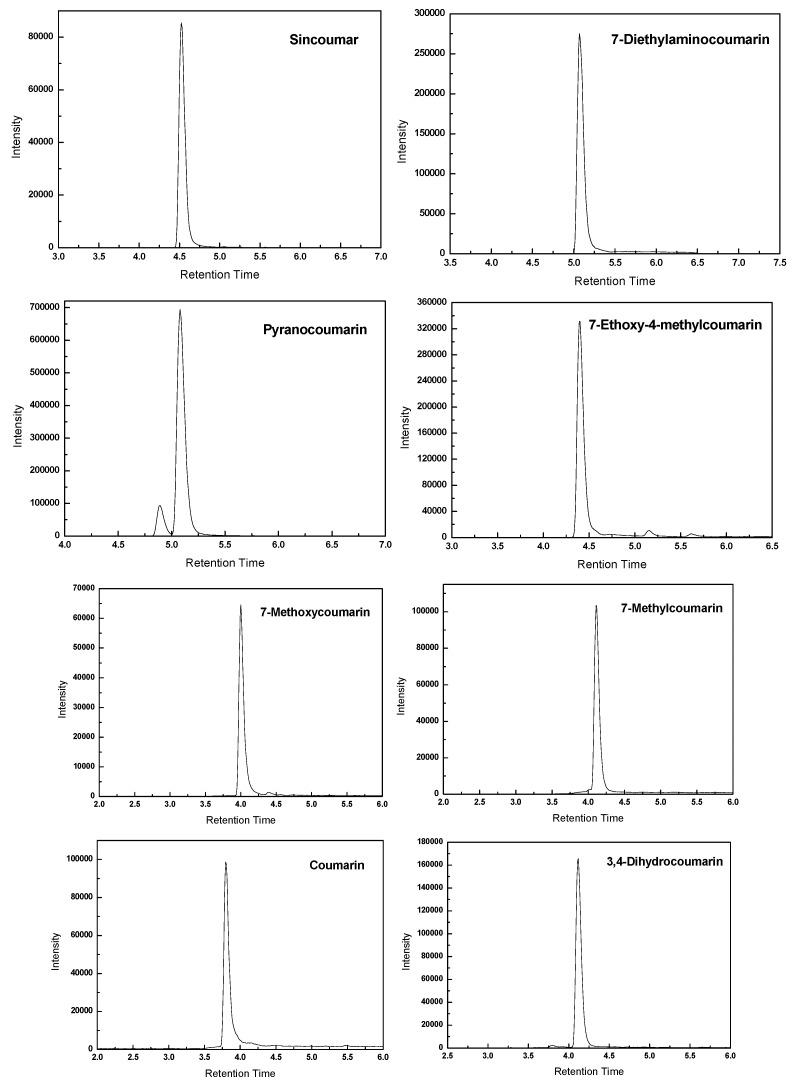
The chromatograms of coumarin and its derivatives spiked at 50 μg·kg^−1^.

The capillary voltage, cone voltage one, cone voltage two, radio frequency voltage lenses, collision energy, mass resolution and other parameters of MS were also optimized to achieve the highest intensity of the analytes. The chromatograms of coumarin and its derivatives spiked at 50 μg·kg^−1^ in MRM mode are shown in Figure 4. It is shown that a good chromatographic separation for coumarin and its derivatives is achieved, providing narrow peaks with good peak symmetry. The high selectivity provided by the MRM mode of the triple quadruple instrument made it possible to separate and quantify all the analytics effectively in a single injection.

### 2.2. Optimization of HPLC Analysis

Some important HPLC details were evaluated in this study. Different C18 columns were used in previous work [31]. Three kinds of C18 columns (Elite Kromasil C18, shimadzu, Osaka, Japan; Waters Symmetry C18, Waters, Port Washington, NY, USA; Agilent Eclipse plus C18, Agilent, Santa Clara, CA, USA) were compared in this paper. Considering the resolution and analysis time, the Agilent Eclipse plus C18 (1.8 μm, 2.1 mm × 50 mm) was chosen as analytical column because of its lower metal impurity content and combination of Waters’ proprietary chemical bonding and end-sealing technology. Three buffer solution (acetonitrile-formic acid in water, methanol-formic acid in water, methanol-formic acid in water) were tested for their HPLC performance, regarding retention time, response and peak shape, and methanol-formic acid in water was selected in this study (data not shown).

### 2.3. Optimization of Extraction and Purification Methods

#### 2.3.1. Optimization of Extraction Solvents

Previous works, such as Yang et al. [32] and Polzin et al. [33], chose methanol as the extraction solvent. Although methanol has good extraction efficiency, it also dissolves many impurities. These studies do not provide much attention to solvent selection. In this study, the recovery of coumarin and its derivatives extracted from tobacco products by six different solvents (acetonitrile, methanol, ethanol, acetonitrile-water (10:90, *v*/*v*), methanol-water (10:90, *v*/*v*), and ethanol-water (10:90, *v*/*v*)) were compared. Acetonitrile showed the best sample recovery among the others (above 81%), therefore, acetonitrile was chosen as the solvent for the extraction process.

#### 2.3.2. Optimization of Purification Material

**Figure 5:**
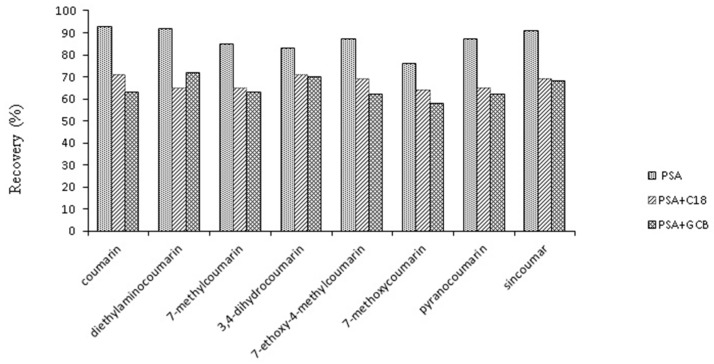
Effect of the different purification sorbents (PSA, C_18_, GCB) on the recovery for coumarin and its derivatives.

In the previously reported studies, purification is a critical procedure for sample preparation for the quantitative determination of flavor additives in tobacco products. The tobacco matrix is very complex due to the presence of high boiling point compounds and dark brown residues which can lower the sensitivity and detection limits. In this study, three different kinds of sample purification sorbents were investigated: Cleanert PSA (50 mg), PSA (25 mg) + C18 (25 mg) and PSA (25 mg) + GCB (25 mg). When the mixed materials PSA + C18 or PSA + GCB, were added to the tobacco extraction solution for purification, the highest recovery of coumarin and its coumarin derivatives was below 72% (Figure 5), while the recoveries of these compounds purified by Cleanert PSA were in the range of 78%–95%, therefore, Cleanert PSA (50 mg) was used as the purification sorbent.

#### 2.3.3. Optimization of Extraction Time

**Figure 6:**
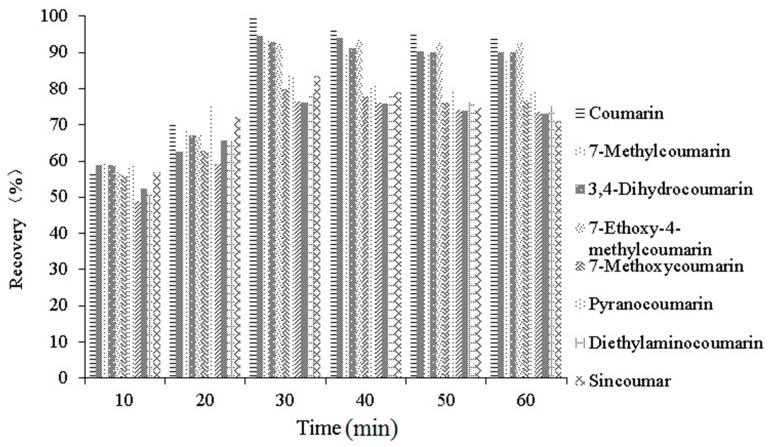
Select the extraction time (10, 20, 30, 40, 50, 60 min).

Extraction time is another important parameter since a short extraction period may lead to incomplete extraction, and an excessively long extraction time may cause changes in the molecular structures of coumarin and its derivatives. Therefore, the effects of different extraction times (10, 20, 30, 40, 50 and 60 min) were investigated. As shown in Figure 6, The recoveries improved slightly from 10 to 20 min, then increased sharply and reached the highest recovery at 30 min (all these compounds have recoveries higher than 70%). The recoveries slowly decreased from 40 to 60 min. Therefore, 30 min of extraction time was used to save time in subsequent experiments.

#### 2.3.4. Optimization of Purification Method

**Figure 7:**
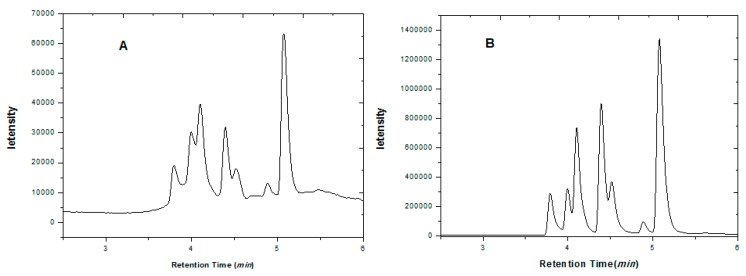
The chromatogram of coumarin and its derivatives before (**A**) and after (**B**) purification by Cleanert PSA.

In general, flavor additives easily adhere to the contents of tobacco and are difficult to extract and purify. In order to extract and purify them effectively, two different pretreatment process were tested. First, 0.5 g tobacco powder was added into a 50 mL of centrifuge tube, then 20 mL of acetonitrile was added. Fifty mg of Cleanert PSA was then added under sonication conditions for 20 min to remove impurities. As the chromatogram in Figure 7A shows, the target substances were not well separated. 

An alternate method was as follows: 0.5 g of tobacco powder was introduced into a 50 mL centrifuge tube. Twenty mL of acetonitrile were added. The extraction was performed under sonication for 30 min. Then the mixture was centrifuged (8000 rpm, 4 °C) for 4 min. Ten mL of the supernatant were carefully transferred to a centrifuge tube, then 50 mg of Cleanert PSA were introduced and the mixture was sonicated for 20 min to remove impurities. As shown in Figure 7B, the eight analytes showed a better separation and the baseline decreased dramatically, so it is very important to remove the matrix impurities and interferences before the purification procedure since this step could improve the elution profile.

### 2.4. Validation

In this study, linearity of calibration curves, limits of detection (LODs), limits of quantitation (LOQs), recoveries and precisions were calculated to demonstrate the validation of the method.

#### 2.4.1. Calibration and Sensitivity

The developed HPLC-MS/MS method validation including linearity, limits of detection (LODs), and limits of quantification (LOQs) was carried out under the optimized condition as shown in Table 2. The calibration curves were based on mean peak area, and the concentrations were set at seven different levels. The calibration curves of the eight analytes were created after the injection (5 μL) of a mixed standard solution. Results showed a good linear relationship over the concentration range studied for each analyte, with correlation coefficients of determination (r^2^) in the range 0.9987–0.9996 seen in Table 2. The LODs of the instrumental method were calculated by the injection of a series of diluted standard solutions until corresponding to a signal-to-noise (S/N) ratio of three. The LOQs were determined by the injection of a series of spiked samples until corresponding to a signal-to-noise (S/N) ratio of ten. Under the optimum condition, the LODs and LOQs of coumarin and its derivatives were in the range of 0.5–1.7 μg·kg^−1^ and 1.7–5.2 μg·kg^−1^, respectively. It is worth mentioning that the LODs of coumarins determined by previous published methods, such as GC-MS [34,35] and HPLC [24,25,36] were around 10–100 μg·kg^−1^, which is much higher than the LODs determined by the HPLC-MS/MS method developed in this study (0.5 μg·kg^−1^). The sensitivity of this method was highly improved over conventional GC methods and the lower LOD makes it possible to determine trace amounts of coumarin compounds in real samples.

#### 2.4.2. Recoveries and Precision

Recovery tests of this validated method were performed in a blank tobacco sample spiked with low (1 × LOQ), intermediate (1.5 × LOQ) and high (2 × LOQ) levels of mixed varied coumarin standards, then the samples were held for 12 hours so that the flavor additives could be thoroughly absorbed before proceeding to extraction and determination. Samples were routinely pretreated and results are summarized in Table 3. The recovery ranges at low, intermediate and high spiked levels were 69.8%–90.5%, 70.4%–93.4% and 72.4%–95.1%, respectively. The recovery levels were acceptable for all eight analytes. In addition, good repeatability of the recovery test (RSD < 5.3%) in all spiked levels was achieved (*n* = 6). Considering all of the above data for method validation, the current HPLC-MS/MS method and sample pretreatment procedures employed in the present work can be regarded as a robust quantification method with a successful application in quantification of the eight different analytes.

### 2.5. Analysis of Real Tobacco Samples

In order to estimate the reliability and practicality of the developed method, samples of thirty five different brands of tobacco purchased at local retail markets were analyzed in this study. Among these thirty five samples, twelve samples contained coumarin or its derivatives and the data are listed in Table 4.

The amounts were given as the average of four determinations. The RSDs of the thirty five tobacco samples ranged from 3.01% to 6.37%. It was clear that the coumarin, 7-methylcoumarin, and 7-ethoxy-4-methylcoumarin were found in most of the twelve samples. 7-Methoxycoumarin and pyranocoumarin were detected in nearly half of the twelve samples, while 3,4-dihydrocoumarin, diethylaminocoumarin and sincoumar were only found in just one sample. The maximum concentration of coumarin and its derivatives were found in sample T7 and the values ranged up to 23.88 mg·kg^−1^. Although these are no reports on the toxic content levels of the seven coumarin derivatives in tobacco, it is worth mentioning that the oral administration of coumarin to mice, rats, and guinea pigs has been reported to give LD_50_ values of 196, 290–680, and 202 mg of coumarin/kg of body weight, respectively [37]. The results verified the usefulness of HPLC-MS/MS for coumarin and its derivatives analysis in tobacco samples. The determination method developed in this study could also help countries seeking to set a maximum admissible concentration of coumarin and its derivatives.

## 3. Experimental

### 3.1. Reagents and Materials

Acetone, methanol and ethanol used were of analytical reagent grade and purchased from Fisher Scientific (Pittsburgh, PA, USA). Water was obtained from a Milli-Q water purification system (Millipore, Redford, MA, USA). Cleanert PSA, C18 and GCB were used in this study (makepolo, Bellefonte, PA, USA). Coumarin, 7-methylcoumarin, 7-methoxycoumarin, 3,4-dihydrocoumarin, 7-ethoxy-4-methylcoumarin, pyranocoumarin, 7-diethylaminocoumarin and sincoumar were purchased from Accustandard (Pittsburgh, CT, USA). Individual stock standard solutions were prepared a concentration of 1000 μg/mL in ethanol and stored at 4 °C. A mixture of all flavor additive standards was prepared by appropriate dilution of individual stock solutions, and stored at 4 °C before use. Flue-cured tobacco leaves prior to cigarette manufacture were used as blank tobacco samples and put into an oven at 40 °C for 4 h to remove moisture and then ground to a 40–60 mesh powder (Kunming Tobacco Plantation, Yunnan, China). Thirty five common brands of cigarettes (29 of Chinese flue-cured tobacco and six of foreign hybrid tobacco) obtained from local retail markets were analyzed in the study. They were kept in a plastic bag and stored in the dark at 4 °C before homogenization and sample preparation.

### 3.2. Sample Treatment

Acetonitrile was chosen to extract the free aroma components in the tobacco products. The tobacco powder was stored in sealed containers and excluded from light. An accurately weighted portion of ground tobacco (approximately 0.5000 g) was put into a 50 mL centrifuge tube. Acetonitrile (20 mL) was added and then samples were extracted under ultrasonic treatment (KQ-500DE CNC sonication cleaner, Shanghai Kedao Ultrasonic Instrument Co.; Ltd., Shanghai, China) for 30 min. After the ultrasonic treatment, samples were centrifuged (Avanti J-26 XPI centrifuge, Beckman Coulter Inc., Redford, MA, USA) for 4 min (8000 rpm, 4 °C) to obtain the supernatant. The supernatant (10 mL) was carefully transferred into a centrifuge tube. Cleanert PSA (50 mg) was added to the tobacco extraction solution under sonication for 20 min to remove impurities. Then the sample solution were concentrated to near dryness by rotary evaporation at 35 °C. The residues were dissolved in 1 mL of methanol-water (10:90, *v*/*v*). Finally 5 μL was used for LC-MS/MS analysis.

### 3.3. HPLC-MS/MS Instrumentation and Conditions

An Agilent 1290 liquid chromatograph (Agilent Technologies, Santa Clara, CA, USA), equipped with a quadruple mass spectrometer (Agilent 6490 tandem mass spectrometer) was used for this study. For the separation, an Agilent Eclipse plus C18 column (1.8 μm, 2.1 mm × 50 mm) equipped with an online filter (Agilent) was used. The mobile phase consisted of 0.1% formic acid (solvent A) and methanol (solvent B). The optimized gradient elution conditions were used as follows: 10%B (0–2 min), 50%B (2.0–2.5 min), 75%B (2.5–3 min), 90%B (3–5 min), 90%B (5–5.5 min), 10%B (5.5–6.5 min). Flow rate was 0.4 mL/min and the column temperature was 35 °C. The sample chamber temperature was 20 °C and the injection volume was 5 μL.

The mass spectrometer MS/MS was equipped with an electrospray ionization (ESI) source. The ESI source was operated in the positive electrospray ionization (ESI^+^) mode. The optimized capillary voltage was set at 3500 V and nebulizer pressure at 137.9 kPa (20 psi). Sheath temperature was kept at 300 °C and sheath gas flow was 11 L·min^−1^. The temperature of drying gas (nitrogen) was 250 °C and its gas flow was 15 L·min^−1^. All qualitative and quantitative data in this study were acquired by using MRM mode where precursors and product ions were monitored simultaneously.

## 4. Conclusions

On the basis of the presented results, the described method using acetonitrile extraction, Cleanest PSA purification, and HPLC-MS/MS quantification is a novel, simple and rapid method for the analysis of coumarin and its derivatives in tobacco products. The proposed method achieved superior selectivity, sensitivity, and accuracy by using MRM mode. Satisfactory recoveries, LODs and LOQs were obtained for the determination of coumarin and its derivatives in tobacco products. The MS/MS fragmentation pathways of coumarin and its derivatives were also elucidated in this paper. Using MRM mode in the proposed method led to superior sensitivity, selectivity, and satisfactory accuracy. The method was successfully applied to real samples and coumarin and its derivatives were detected in real tobacco products. The results demonstrated the potential of the HPLC-MS/MS method for the routine analysis of coumarin derivatives flavor additives in tobacco products. This method could allow governments to establish relevant regulations.

## Figures and Tables

**Table 1:** Elemental composition, retention time, MS/MS parameters for coumarin and its derivatives (* Quantitation ion pair).

Compound	CAS	Elemental Composition	Retention Time (min)	Ionization Mode	Precursor (*m*/*z*)	Production (*m*/*z*) (Collision Energy/eV)
Coumarin	91-64-5	C_9_H_6_O_2_	3.79	[M + H]^+^	147.3	91.0 * (25); 103.1 (15)
7-Methylcoumarin	2445-83-2	C_10_H_8_O_2_	4.10	[M + H]^+^	161.1	105.0 * (25); 115.1 (20)
3,4-Dihydrocoumarin	119-84-6	C_9_H_8_O_2_	4.10	[M + H]^+^	149.1	107.0 * (10); 7 7.0 (15)
7-Ethoxy-4-methyl-coumarin	87-05-8	C_12_H_12_O_3_	4.39	[M + H]^+^	205.1	177.2 * (20); 105.0 (25)
7-Methoxycoumarin	531-59-9	C_10_H_8_O_3_	3.99	[M + H]^+^	177.1	121.0 * (25); 132.9 (15)
Pyranocoumarin	518-20-7	C_20_H_18_O_4_	5.08	[M + H]^+^	323.1	251.0 * (15); 291.0 * (10)
7-Diethylaminocoumarin	63226-13-1	C_27_H_28_N_2_O_5_	5.08	[M + H]^+^	461.0	244.0 * (25); 417.0 (35)
Sincoumar	152-72-7	C_19_H_15_NO_6_	4.52	[M + H]^+^	354.1	296.1 * (20); 162.9 (15)

**Table 2:** Linear range, correlation coefficient, limits of detection (LOD) and limits of quantification (LOQ) for coumarin and its derivatives.

Analytes	Linear Range (μg·kg^−1^)	Regression Equation	Correlation Coefficient (r^2^)	LOD	LOQ
				(μg·kg^−1^)	(μg·kg^−1^)
Coumarin	2–500	*Y* = 10493*X* + 26.32	0.9987	0.5	2.0
7-Methylcoumarin	5–500	*Y* = 16661*X* + 24.99	0.9989	0.9	3.0
3,4-Dihydrocoumarin	5–500	*Y* = 17936*X* + 152.4	0.9996	1.5	5.0
7-Ethoxy-4-methylcoumarin	2–500	*Y* = 37567*X* + 94.14	0.9995	0.5	1.7
7-Methoxycoumarin	5–500	*Y* = 19204*X* + 86.41	0.9993	1.2	3.5
Pyranocoumarin	5–500	*Y* = 71867*X* + 21.56	0.9992	0.6	2.1
7-Diethylaminocoumarin	5–00	*Y* = 32635*X* + 44.76	0.9994	1.5	5.0
Sincoumar	5–500	*Y* = 10161*X* + 45.72	0.9995	0.9	3.1

**Table 3:** Recovery and precision of the investigated compounds.

NO.	Analytes	Spiked Level (μg·kg^−1^)	Average Recovery (%)	RSD (%)
1	Coumarin	2.0	76.1	3.2
		3.0	85.0	2.1
		4.0	88.3	1.5
2	7-Methylcoumarin	3.0	75.1	5.3
		4.5	78.4	2.4
		6.0	76.3	2.0
3	3,4-Dihydrocoumarin	5.2	69.8	5.3
		7.8	70.9	4.4
		10.4	72.4	2.5
4	7-Ethoxy-4-methylcoumarin	1.7	86.8	2.2
		2.5	91.3	2.1
		3.4	94.2	2.2
5	7-Methoxycoumarin	3.5	90.5	3.7
		5.3	93.4	2.7
		7.0	95.1	1.9
6	Pyranocoumarin	2.1	81.5	4.3
		3.2	90.0	3.3
		4.2	94.3	2.3
7	7-Diethylaminocoumarin	5.0	73.2	3.4
		7.5	76.5	3.8
		10.0	81.2	2.9
8	Sincoumar	3.1	80.5	5.3
		4.7	84.3	4.2
		6.2	83.5	3.7

**Table 4:** Coumarin and its derivatives in commercial tobacco samples (mg·kg^−1^, *n* = 4).

Compounds	T1	T2	T3	T4	T5	T6	T7	T8	T9	T10	T11	T12
Coumarin	5.4	3.51	5.6	5.21	4.72	2.35	5.67	3.2	-	4.38	5.16	2.77
7-Methylcoumarin	4.9	1.87	3.71	2.89	3.01	1.61	4.32	-	1.93	-	1.89	2.17
3,4-Dihydrocoumarin	-	-	-	-	-	-	5.21	-	-	-	-	-
7-Ethoxy-4-methylcoumarin	3.72	3.61	3.95	2.99	3.14	2.13	1.81	3.91	2.07	2.64	3.45	3.05
7-Methoxycoumarin	-	3.53	-	3.75	-	-	3.53	-	3.55	-	3.25	-
Pyranocoumarin	-	2.29	-	2.23	-	2.45	-	2.53	-	2.42	2.31	-
Diethylaminocoumarin	-	5.48	-	-	-	-	-	-	-	-	-	-
Sincoumar	-	-	-	-	-	-	3.34	-	-	-	-	-
**Total coumarins content**	**14.02**	**20.29**	**13.26**	**17.07**	**10.87**	**8.54**	**23.88**	**9.64**	**7.55**	**9.44**	**16.06**	**4.94**

-: The content is lower than LOD.
